# Can Defective Schreibersite Deliver Prebiotic Phosphorus?

**DOI:** 10.1021/acsearthspacechem.6c00158

**Published:** 2026-09-07

**Authors:** Stefano Pantaleone, Albert Rimola, Nadia Balucani, Piero Ugliengo

**Affiliations:** † Dipartimento di Chimica and Nanostructured Interfaces and Surfaces (NIS) Centre, 9314 Università degli Studi di Torino, via P. Giuria 7, Torino I-10125, Italy; ‡ Departament de Química, 16719 Universitat Autònoma de Barcelona, Bellaterra, Catalonia 08193, Spain; § Accademia delle Scienze di Torino, Torino 10123, Italy; ∥ Dipartimento di Chimica, Biologia e Biotecnologie, 9309 Università degli Studi di Perugia, Via Elce di Sotto 8, Perugia I-06123, Italy; ⊥ Osservatorio Astrofisico di Arcetri, Largo E. Fermi 5, Firenze I-50125, Italy; # Université Grenoble Alpes, CNRS, Institut de Planétologie et d’Astrophysique de Grenoble (IPAG), Grenoble F-38000, France

**Keywords:** DFT, periodic surface, defects, corrosion, prebiotic chemistry

## Abstract

The origin of bioavailable phosphorus remains one of the major unresolved questions in prebiotic chemistry. The most widely accepted hypotheses invoke an exogenous delivery of phosphorus during the Archean Eon (ca. 4 billion years ago), associated with the Late Heavy Bombardment and involving the iron–nickel phosphide mineral schreibersite ((Fe,Ni)_3_P). Over the past decades, these hypotheses have been supported by aqueous corrosion experiments, showing that schreibersite releases a variety of oxygenated phosphorus species, including phosphates. In this work, we investigate the wetting and corrosion of the reactive schreibersite (001) surface (Fe_2_NiP) by means of periodic DFT simulations up to the formation of phosphate species. Unlike previous studies, which considered only the pristine (001) surface, here we investigate defective surface models to assess structural defects that facilitate phosphate formation. The results reveal significant changes in the thermodynamics of the corrosion process, whereas the kinetic barriers remain remarkably similar to those calculated for the pristine (001) surface, differing by less than 5 kJ mol^–1^, well within the intrinsic chemical accuracy of DFT calculations. These findings indicate that the pristine (001) surface provides a reliable and robust structural model for investigating phosphate formation and, potentially, more complex prebiotic processes, such as phosphorylation of organic molecules. Consequently, increasingly complex structural models representing advanced stages of corrosion are not required, despite the substantial morphological and compositional changes expected to occur during surface degradation.

## Graphical abstract

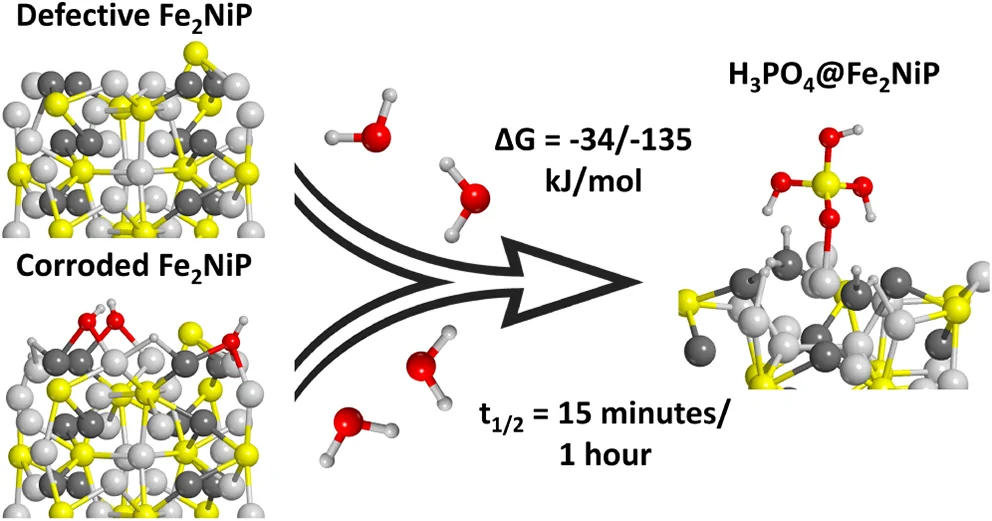

## Introduction

Phosphorus is one of the six essential macroelements of life, together with hydrogen, carbon, nitrogen, oxygen, and sulfur. Although relatively scarce in living organisms, phosphorus plays a ubiquitous role in biology, primarily in the form of inorganic phosphate and organophosphate groups. At physiological pH, inorganic phosphate exists predominantly as the conjugate acid–base pair H_2_PO_4_^–^/HPO_4_^2–^. Phosphate groups perform numerous essential biological functions, including serving as the energy currency in ATP, forming the polar headgroups of membrane phospholipids, and constituting the structural backbone of DNA and RNA.^1^

The prebiotic origin remains uncertain. In terrestrial P-bearing minerals, phosphorus is present almost exclusively as phosphate (PO_4_^3–^), a highly oxidized and poorly soluble species that is strongly bound within mineral matrices and is therefore largely unavailable for the phosphorylation of prebiotic building blocks such as sugars, lipids, and nucleosides.^2^ One of the earliest solutions to this problem came from Gulick, who identified the meteoritic iron–nickel phosphide mineral schreibersite ((Fe,Ni)_3_P)^3,4^ as a potential source of reduced and chemically reactive phosphorus.^5^ Over the past few years, both computational studies and laboratory experiments have supported the hypothesis that reduced phosphorus was continuously supplied to the early Earth, either through extraterrestrial delivery of schreibersite^6–9^ or through its in situ formation following lightning strikes on clay-rich soils.^10^ The latter mechanism has recently been proposed as a major endogenous source of reactive phosphorus because of the high frequency of lightning during the Archean Eon. The susceptibility of schreibersite to aqueous corrosion was experimentally demonstrated by Pasek and coworkers in 2005,^11^ who showed that its reaction with water produces not only phosphates but, more importantly, a variety of reactive oxyphosphorus intermediates, including polyphosphates and phosphorus–oxygen radicals capable of phosphorylating biologically relevant organic molecules such as sugars and nucleotides.^12–14^

The atomistic mechanism of the schreibersite corrosion process has been the subject of recent studies, which show that, even under mild conditions (room temperature and atmospheric pressure), schreibersite reacts with water and methanol, initiating the corrosion of the material.^15–17^ From the computational standpoint, a few works were devoted to the bulk structure of schreibersite, in order to study the effect of the pressure (in the interior of meteorites or during the meteor impact) on the stability of the different phases.^18–20^ More recently, other studies focused on the adsorption and reaction of water on crystalline bare surfaces,^21,22^ confirming the endothermic deprotonation of water, up to the proposal of simplified reaction pathways that, despite all the approximations in the methodology and the structural models, provide insights into the feasibility of this process already at room temperature, and without external energy sources.^23–25^

In this work, we focus on two different defective models of the reactive (001) schreibersite surface, to account for one of the key aspects that makes surface corrosion reactions more complex than idealized systems: i.e., the surface structure continuously changes as the reaction proceeds and, accordingly, the mechanism as well. The aim of this work is to compare these defective surface models with our previously investigated pristine (001) surface in order to assess whether structural defects significantly affect the later stages of the corrosion process.

## Computational Details

The defective Schreibersite surfaces and their interaction with water were investigated using periodic DFT calculations as implemented in the Vienna Ab-initio Simulation Package (VASP) code,^26–29^ which uses projector-augmented wave (PAW) pseudopotentials^30^ to describe the ionic cores and a plane wave basis set for the valence electrons.

Geometry optimizations and vibrational frequency calculations were performed using the gradient-corrected PBE exchange-correlation functional,^31^ together with a posteriori Grimme D2 dispersion correction,^32^ as modified for solids (D*).^33^ In this case, the C6 atomic coefficients related to polarizabilities on Fe and Ni metal atoms were set to 0 (i.e., no dispersion interaction contribution from metal atoms). This computational setup was adopted because it provided the best agreement with structural and experimental spectroscopic properties as reported in our previous work on the bulk and bare surfaces of Schreibersite.^21^ The original D* parameters were retained for oxygen and hydrogen atoms. This computational setup is hereafter referred to as PBE-D*0. The plane-wave kinetic-energy cutoff was set to 500 eV. The self-consistent field (SCF) iterative procedure was set to a tolerance in total energy of ΔE = 10^–5^ eV for geometry optimizations of minima, while for frequency calculations the tolerance was decreased to ΔE = 10^–6^ eV. For transition state searches the NEB method was used,^34–38^ and the structures were then refined with the DIMER method:^39–42^ in this case the SCF was converged to ΔE = 10^–7/–8^ eV to ensure that the algorithm correctly identifies and follows the negative curvature of the PES toward the saddle point. The tolerance on gradients during the optimization procedure was set to 0.01 eV/Å for each atom in each direction. During geometry optimization, all atoms in the unit cell were allowed to relax whereas the lattice parameters were kept fixed at the optimized geometry of the bulk structure (a = 8.984 Å, b = 8.984 Å, γ = 90.000°).^21^ A Monkhorst–Pack k-point mesh of (4 4 1) was adopted, the last one referring to the nonperiodic direction (i.e., no sampling of the reciprocal space). Since plane-wave calculations employ periodic boundary conditions, the slab is periodically replicated along the surface normal. A vacuum layer of at least 20 Å was therefore introduced to minimize spurious interactions between periodic images.

Vibrational frequencies were calculated at Γ point, by numerical differentiation of the analytical first derivatives, using the central difference formula (i.e., two displacements of 0.015 Å for each atom in each (x, y, z) direction), in order to confirm that the optimized structure is a minimum (all real frequencies) or a transition state (one imaginary frequency). Only H and O atoms were allowed to vibrate, while the remaining ones are kept fixed to save computing time. The accuracy of this approach has previously been validated against full Hessian calculations (see Tables S2 and S3 in ref 25). Thermochemistry has been corrected using the quasi-harmonic approximation, proposed by Grimme,^43^ in which frequencies below 100 cm^–1^ were treated as free internal rotations. This improves the estimation of thermal corrections, which would be otherwise underestimated when considering very low frequency values. To avoid discontinuity close to the cutoff, a damping function was used to interpolate the values computed within the two ranges of frequencies.

The rate constant of water dissociation, was calculated using the Eyring equation assuming a unimolecular process:^44,45^

1 $$k=\frac{k_{B}T}{h}e^{\frac{\Delta G^{‡}}{RT}}$$

where *k*_B_ is the Boltzmann constant, *T* is the absolute temperature, *R* is the ideal gas constant, *h* is the Planck constant and *ΔG* ^‡^ is the difference of free energy between the transition state and the preceding minimum. This treatment is based on two assumptions: (1) water is available in excess, and (2) the energy released upon adsorption is rapidly dissipated into the schreibersite lattice, preventing it from significantly influencing the reaction kinetics.^46,47^ Under these assumptions, the subsequent elementary steps involving the adsorbed water molecule (deprotonation and hydroxyl migration) can be treated as unimolecular surface reactions, within a pseudo-first-order approximation for the estimation of the reaction half-life time:

2 $$t_{1/2}=\frac{\ln2}{k}$$

Structural visualization and manipulation and figure rendering were performed using the MOLDRAW,^48^ VMD,^49^ and POV-Ray.^50^

## Results

### Surface Models of Advanced Corroded Stages

Figure 1 compares the pristine (001) schreibersite surface (Figure 1a) with two defective models designed to represent progressively more advanced stages of the corrosion process.

**Figure 1:**
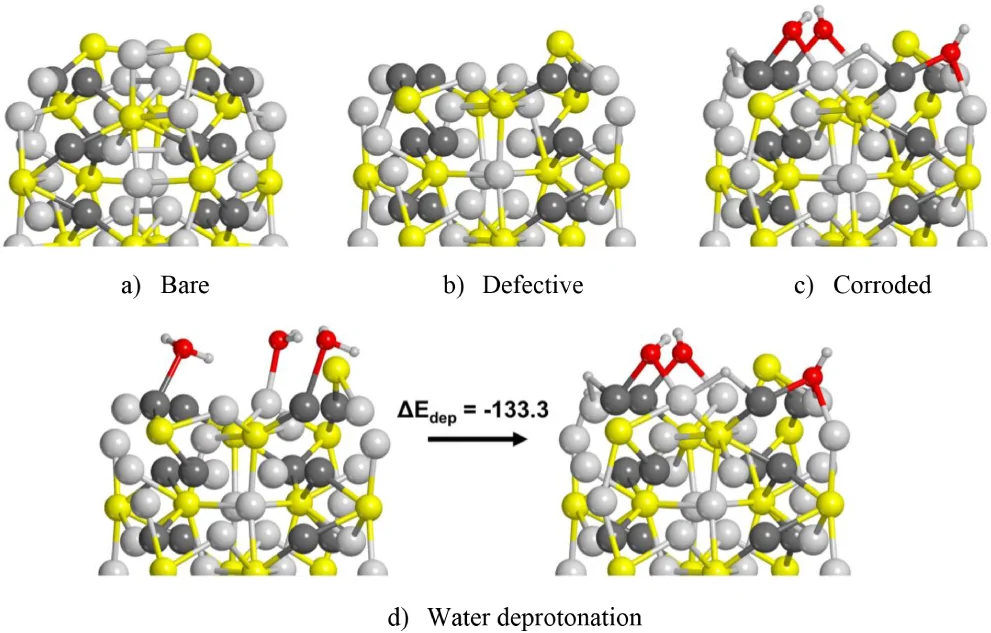
PBE-D*0 optimized structures of different (001) schreibersite surfaces: (a) as extracted from the bulk structure, (b) after removal of 1 P and 2 Fe atoms, (c) as (b) with addition of 3 dissociated H_2_O. (d) Water deprotonation reaction: ΔE_dep_ is the total deprotonation energy (in kJ/mol), i.e., not normalized per water molecule. Atom color legend: H in white, O in red, P in yellow, Fe in light gray, Ni in dark gray.

Specifically, Figure 1b shows a defective surface where one phosphorus atom and two iron atoms were removed from the bare surface (hereafter called “defective”), thereby generating an electrically neutral, nonstoichiometric surface. The model was designed to leave a single exposed phosphorus atom, which serves as the reactive center for investigating the migration of hydroxyl groups, originating from a previous water dissociation step, leading to the formation of the phosphate.

The second model (Figure 1c) was generated from the defective surface by adsorbing three dissociated water molecules atop Fe and Ni atoms, near the exposed P atom (hereafter referred to as “corroded”): the number of water molecules was chosen according to the maximum number of adsorption sites to accommodate dissociated water molecules. Indeed, in Figure 1d we show that the deprotonation of three water molecules is energetically favorable (ΔE_dep_ = −133.3 kJ/mol, −44.4 kJ/mol per water molecule). At this stage, the surface becomes too crowded to host the deprotonation of a fourth water molecule.

The idea was to simulate an advanced stage of corrosion, in which the first layer of atoms of the surface has already been consumed by water molecules and detached from the underlying bulk as iron and nickel oxides and hydroxides, in a process analogous to rust formation on metallic iron. The two models can be regarded as representing different abundances of water in the environment: the defective model represents microsolvation phenomena, possibly occurring to the material exposed to the atmosphere, while the corroded model would represent the contact of schreibersite with liquid water. We do not intend these models to represent a general description of a corroded surface. Rather, they were designed solely to reproduce the same reaction pathway previously investigated on the pristine (001) schreibersite surface, allowing us to assess whether the choice of the initial structural model has any significant impact on the thermodynamics or kinetics of the corrosion process.

### Reactions Paths: From H_2_O to H_3_PO_4_

Figures 2 and 3 show the water corrosion process on defective (001) schreibersite. The reaction proceeds through successive adsorption of water on a metal center (Fe or Ni), its dissociation and successive migration toward P, following the same mechanism observed to the pathway on the bare (001) schreibersite surface.^25^ Tables S1–S3 report the full reaction path of the water corrosion on the three (001) schreibersite surface models for comparison. In some cases, a few additional elementary steps are needed, reach the appropriate intermediate geometry to form the subsequent intermediate. An example is the deprotonation of the P­(OH)_n_ group (1H_2_O-POH-DEPT-TS in Figures 2 and 4 H_2_O-POH_3_-DEPT-TS in Figure 3), in all cases associated with a substantial thermodynamic stabilization ( $\Delta G^{1H_{2}O‐PO‐H‐H-1H_{2}O‐POH}=-85.2-20.1=-105.3kJ/mol$ , and $\Delta G^{4H_{2}O‐POH_{3}‐DEPT-4H_{2}O‐DEPT}=-149.5-(-110.4=-39.1kJ/mol)$ ), at a negligible energy barrier, with respect to the other barriers ( $\Delta G_{1H_{2}O‐POH‐DEPT‐TS}^{‡}=5.9kJ/mol$ and $\Delta G_{1H_{2}O‐POH‐DEPT‐TS}^{‡}=26.6kJ/mol$ ). Another example involves conformational changes which, however, do not affect either the thermodynamics or the kinetics of the process (1H_2_O–OH-ROT-TS in Figure 2). Among all elementary processes, the rate-determining steps consistently correspond to hydroxyl migration from the metal center to the phosphorus atom, with activation free energies ranging from 74.1 (3H_2_O-DIFF-TS) to 106.8 (1H_2_O-OH-DIFF-TS) kJ/mol and endothermic reaction Gibbs energies ( $\Delta G^{1H_{2}O‐POH-1H_{2}O‐OH‐ROT}=-20.1-\left(-80.6\right)=60.5kJ/mol$ , and $\Delta G^{2H_{2}O‐PO‐OH-2H_{2}O‐DEPT}=-79.5-\left(-165.1\right)=85.6kJ/mol$ ). This trend is consistent with our previous studies which established the following order of surface reactivity: the most reactive adsorption and deprotonation centers are the metals (if Fe or Ni it depends on the surface and the exposure to the vacuum of the selected site), phosphorus atoms are poor adsorption sites for molecular water, while water deprotonation occurs endothermically, at variance with Fe and Ni atoms.^22^ This behavior is partially compensated, even if it does not revert, when more stable thermodynamic species are formed (in a terrestrial environment), i.e., POH_3_ ( $\Delta G^{3H_{2}O‐POH_{3}-3H_{2}O‐DEPT}=-46.0-\left(-58.0\right)=12.0kJ/mol$ ) and H_3_PO_4_ ( $\Delta G^{4H_{2}O{‐H}_{3}PO_{4}-4H_{2}O‐POH_{3}‐DEPT}=-134.8-\left(-149.5\right)=14.7kJ/mol$ ). Overall, the global process is strongly exothermic ( $\Delta G_{4H_{2}O{‐H}_{3}PO_{4}}=-134.8kJ/mol$ ), and the kinetic constant, calculated on the highest activation barrier ( $\Delta G^{‡}G_{4H_{2}O{‐H}_{3}PO_{4}}=106.8kJ/mol$ ) at room temperature (298 K), is equal to 1.18 × 10^–6^ s^–1^, which corresponds to a half-life time, assuming a first order kinetics, of 163 h, which becomes ca. 15 min when the temperature slightly increases up to 350 K. It should be noted that these are often shock processes, involving meteor impacts which can increase the temperature up to 2000 K, making the reaction instantaneous (within a few picoseconds).

**Figure 2:**
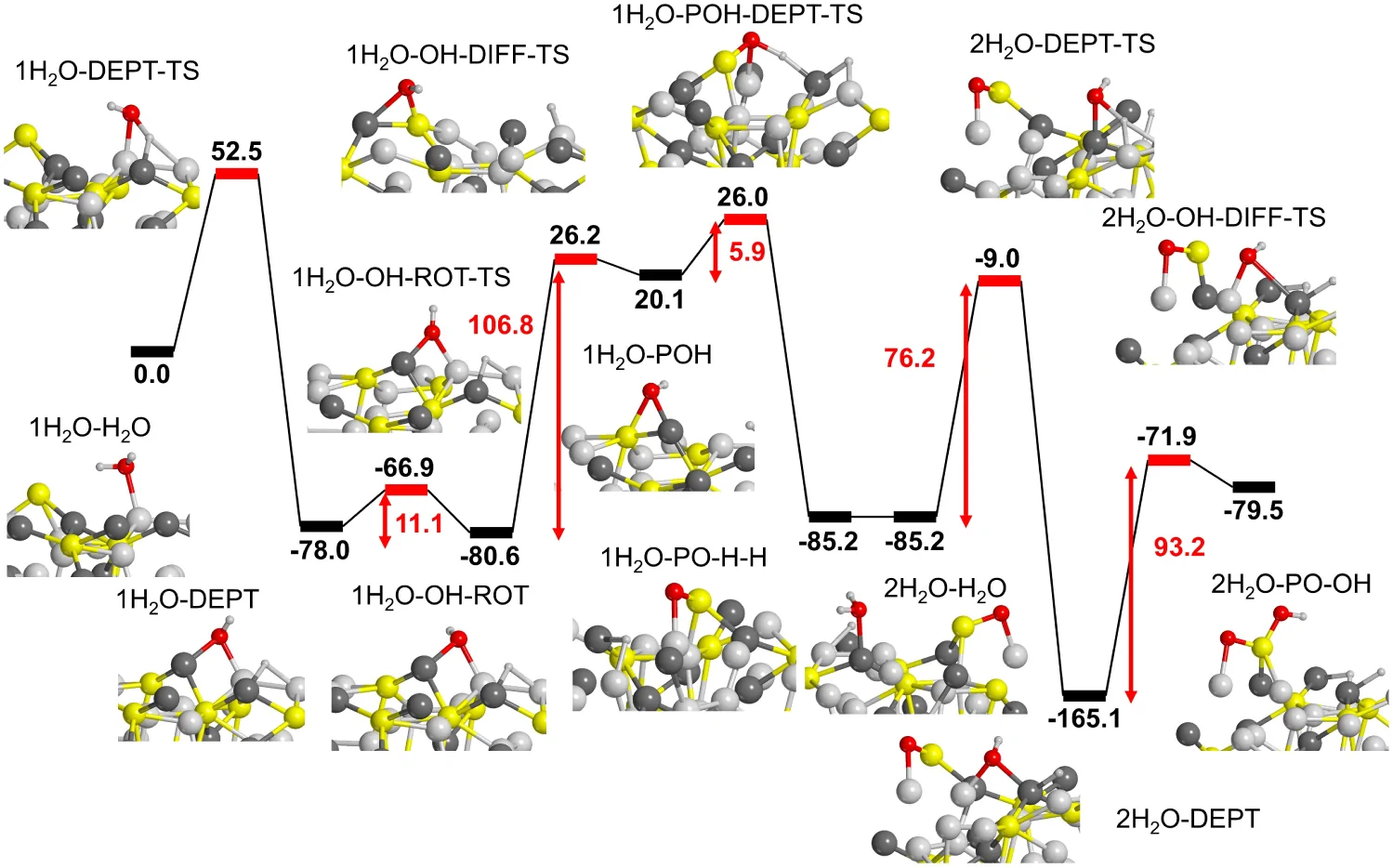
PBE-D*0-Gibbs energy reaction pathway at 298 K (in kJ/mol) of the water corrosion process on defective (001) schreibersite surface for the first and second water molecules. Atom color legend: H in white, O in red, P in yellow, Fe in light gray, Ni in dark gray. Transitions states and activation barriers are highlighted in red.

**Figure 3:**
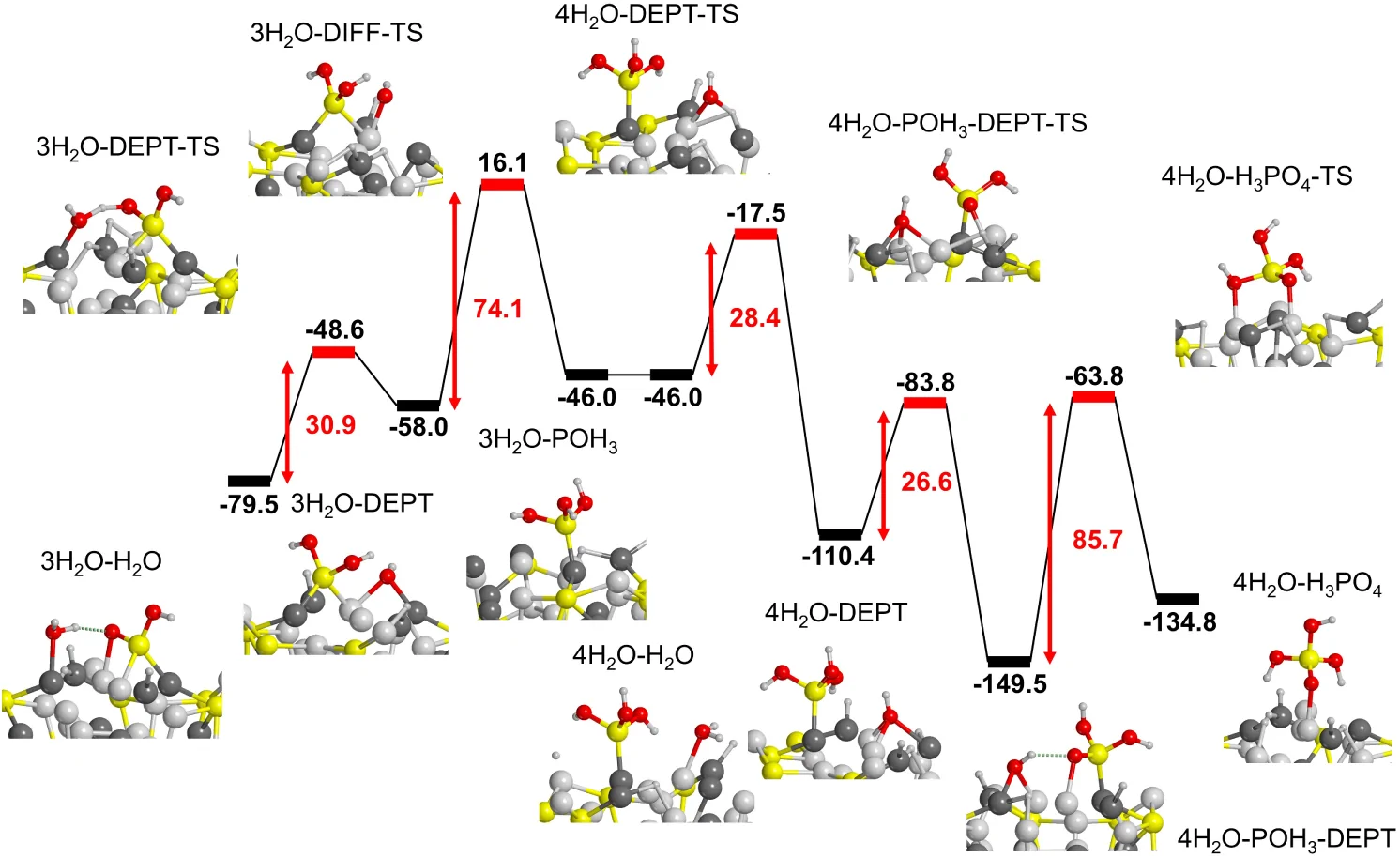
PBE-D*0-Gibbs energy reaction pathway at 298 K (in kJ/mol) of the water corrosion process on defective (001) schreibersite surface for third and fourth water molecules. Atom color legend: H in white, O in red, P in yellow, Fe in light gray, Ni in dark gray. Transitions states and activation barriers are highlighted in red.

**Figure 4:**
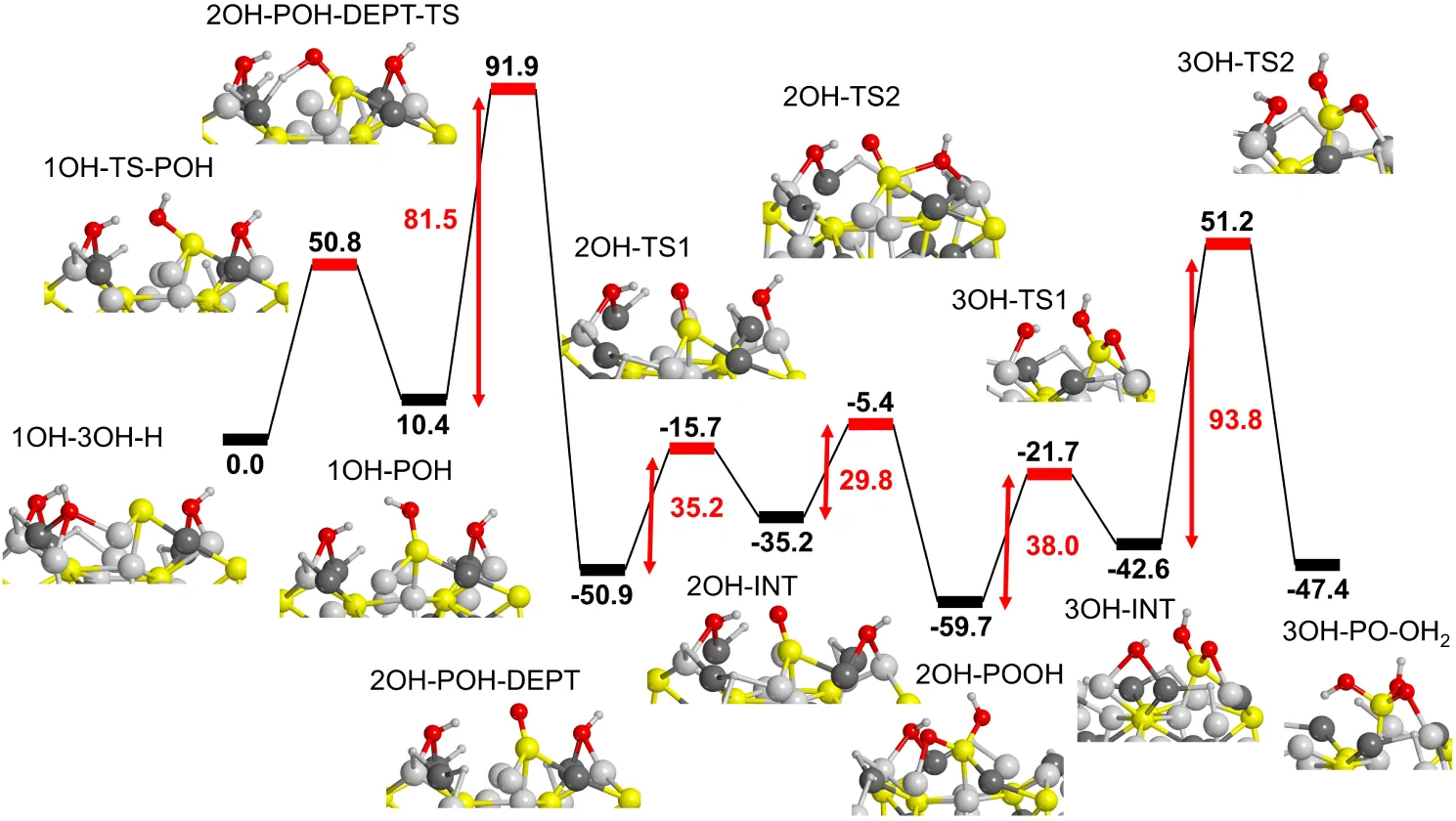
PBE-D*0-Gibbs energy reaction pathway at 298 K (in kJ/mol) of the water corrosion process on corroded (001) schreibersite surface for first to third water molecules. Atom color legend: H in white, O in red, P in yellow, Fe in light gray, Ni in dark gray. Transitions states and activation barriers are highlighted in red.

Figures 4 and 5 show the water corrosion process on corroded (001) schreibersite. In this case, the range of the activation barriers associated with hydroxyl diffusion is wider, from 29.8 to 111.4 kJ/mol; the rate constant of the rate-determining step at 350 K increases to 1.73 × 10^–4^ s^–1^ (see eq 1) corresponding to a half-life time of about 1 h (see eq 2). The global thermodynamics of the reaction is slightly less favorable than that on the defective surface ( $\Delta G_{4H_{2}O{‐H}_{3}PO_{4}}=-134.8kJ/mol$ and $\Delta G_{4OH{‐H}_{3}PO_{4}}=-34.1kJ/mol$ ). The value reported in our previous study for the same reaction on the pristine (001) schreibersite surface falls between those obtained for the two defective models investigated here (ΔG = −69 kJ mol^–1^). Figure 6 summarizes the key quantities used to compare the three reaction pathways, namely the highest activation barriers and the free energy of the final minimum, corresponding to phosphate formation. As already demonstrated in previous papers, water dissociation is strongly exothermic, especially during adsorption of the first molecules;^23^ however, in the corroded (001) schreibersite we started corrosion with already dissociated waters, without therefore taking into account this extra-stabilization. Nevertheless, the reaction is still slightly exothermic, indicating that the formation of the phosphate is favorable, despite the substantial structural changes introduced in the initial surface model.

**Figure 5:**
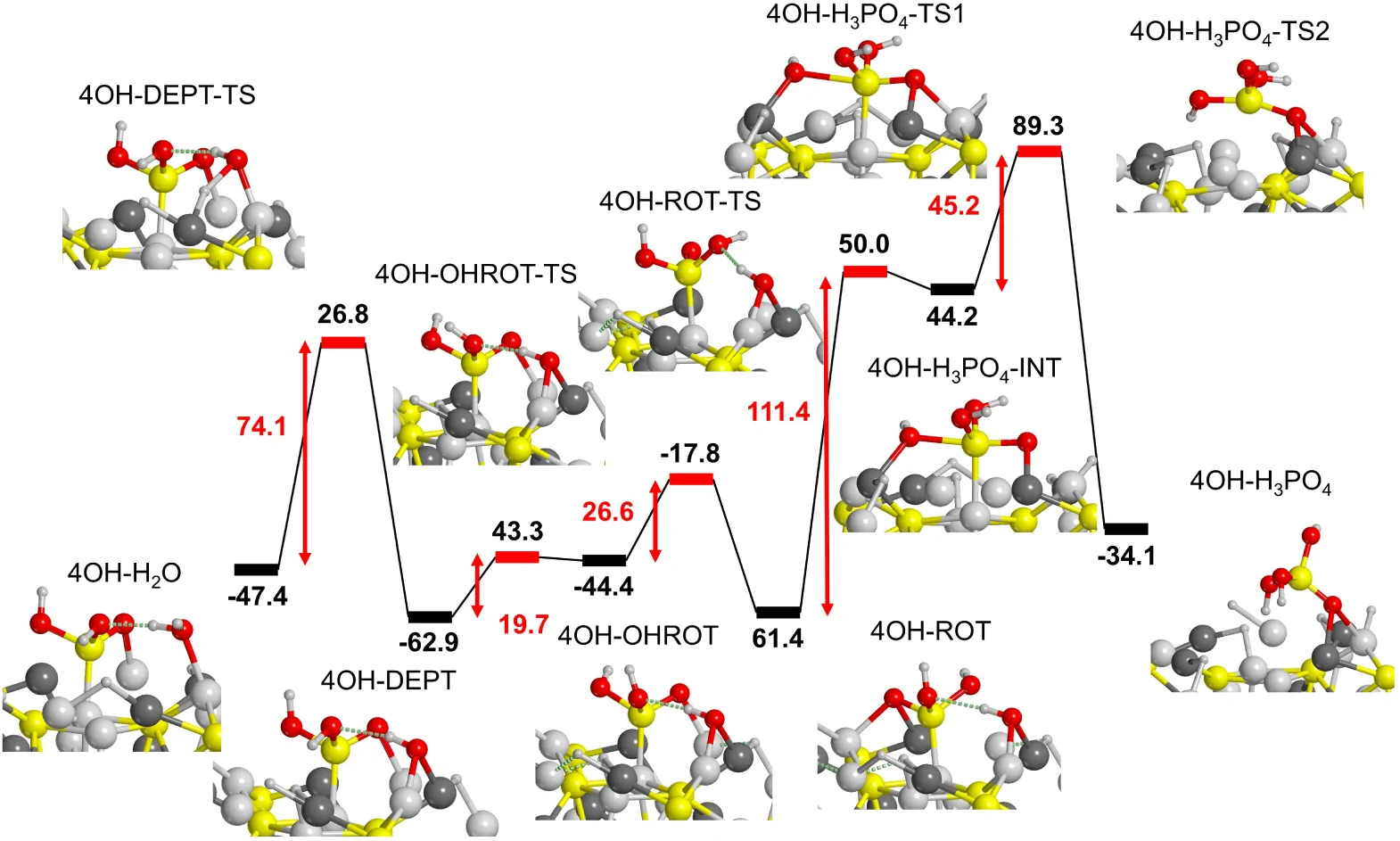
PBE-D*0-Gibbs energy reaction pathway at 298 K (in kJ/mol) of the water corrosion process on the corroded (001) schreibersite surface for the fourth water molecule. Atom color legend: H in white, O in red, P in yellow, Fe in light gray, Ni in dark gray. Transitions states and activation barriers are highlighted in red.

**Figure 6:**
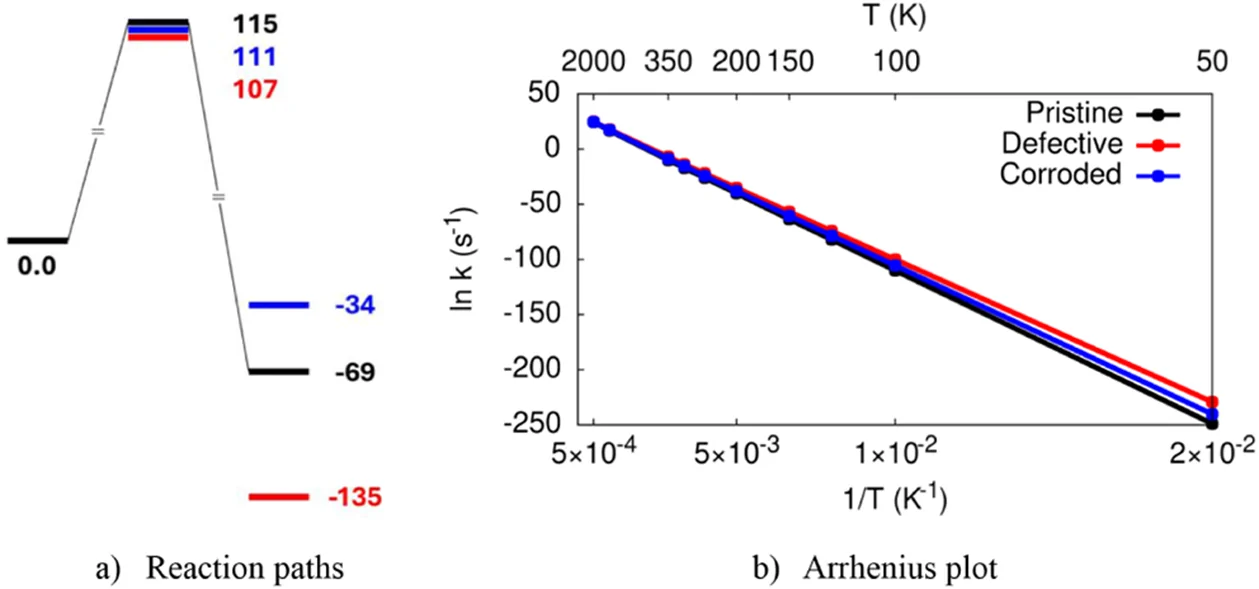
(a) Summary of the reaction pathway, showing only the highest activation barriers and the final minima. (b) Rate constant (calculated from the highest activation barrier) as a function of temperature. Black denotes the pristine (001) surface, as reported in ref 25, whereas red and blue correspond to the defective and corroded models investigated in this work.

Finally, Figure 6 summarizes the reaction pathways calculated for the water-induced corrosion of the three schreibersite surface models, namely the pristine, defective, and corroded surfaces. Panel (a) compares the two key energetic quantities governing the overall process, namely the highest activation free energy and the overall Gibbs free energy change leading to the final product (phosphate). Although the overall reaction free energy differs quantitatively among the three models (ΔG = −69, −135, and −34 kJ mol^–1^ for the pristine, defective, and corroded surfaces, respectively), all reaction pathways remain thermodynamically favorable. These quantitative differences are expected because each model represents a substantially different initial surface configuration. In contrast, the kinetics are remarkably similar. The highest activation barriers, corresponding to the rate-determining steps, are 115, 107, and 111 kJ mol^–1^ for the pristine, defective, and corroded models, respectively. In all cases, the rate-determining step involves hydroxyl migration from a metal adsorption site (typically bridging Fe–Ni or Fe–Fe pairs) to the phosphorus atom. Panel (b) reports the Arrhenius plots associated with the rate-determining step for the three reaction pathways, while Table 1 reports the rate constants and the half-life times of the three reactions at all considered temperatures. It should be noted that the highest activation barrier does not necessarily correspond to the same elementary reaction. Indeed, for the pristine and corroded surfaces, the rate-determining step is the formation of the phosphate product, whereas for the defective surface it corresponds to the formation of the first P–OH bond.

**Table 1:** Rate Constant (in s^–1^) and Corresponding Half-Life Times (in Hours), of the Highest Activation Barriers for the Reaction on the Three Surfaces

	Pristine		Defective		Corroded	
T	k	*t*_1/2_	k	*t*_1/2_	k	*t*_1/2_
50	7.59 × 10^–109^	2.54 × 10^104^	2.80 × 10^–100^	6.88 × 10^95^	4.38 × 10^–105^	4.40 × 10^100^
100	1.78 × 10^–48^	1.08 × 10^44^	3.41 × 10^–44^	5.64 × 10^39^	1.35 × 10^–46^	1.43 × 10^42^
125	2.30 × 10^–36^	8.39 × 10^31^	6.13 × 10^–33^	3.14 × 10^28^	7.33 × 10^–35^	2.63 × 10^30^
150	2.81 × 10^–28^	6.84 × 10^23^	2.02 × 10^–25^	9.55 × 10^20^	5.04 × 10^–27^	3.82 × 10^22^
200	3.85 × 10^–18^	5.00 × 10^13^	5.33 × 10^–16^	3.61 × 10^11^	3.36 × 10^–17^	5.74 × 10^12^
250	4.89 × 10^–12^	3.94 × 10^07^	2.53 × 10^–10^	7.62 × 10^05^	2.76 × 10^–11^	6.97 × 10^06^
298	4.32 × 10^–08^	4.45 × 10^03^	1.18 × 10^–06^	1.63 × 10^02^	1.85 × 10^–07^	1.04 × 10^03^
350	5.02 × 10^–05^	3.84 × 10^00^	8.40 × 10^–04^	2.29 × 10^–01^	1.73 × 10^–04^	1.11 × 10^00^
1000	2.05 × 10^07^	9.39 × 10^–12^	5.50 × 10^07^	3.50 × 10^–12^	3.16 × 10^07^	6.09 × 10^–12^
2000	4.13 × 10^10^	4.66 × 10^–15^	6.77 × 10^10^	2.84 × 10^–15^	5.13 × 10^10^	3.75 × 10^–15^

## Conclusions

In this work, periodic DFT calculations were employed to investigate the corrosion of the schreibersite (001) surface by water, following the reaction mechanism up to phosphoric acid formation. The reactive (001) surface was considered, as already done in our previous paper,^25^ to enable direct comparison of previous results. In this case, instead of the bare surface, we started from two representative models of advanced corrosion stages, which can be considered as model structures of advanced stages of corrosion: one (called “defective”) in which Fe and Ni outermost atoms were removed, thus taking only the most exposed P atom, and another one (called “corroded”), where dissociated H_2_O molecules were adsorbed on the “defective” model. Both defective models exhibit reaction energetics remarkably similar to those obtained for the pristine surface (see Figure 6a): The similarity of the previous results indicates that, although surface corrosion is intrinsically complex to simulate, as the structure of the surface continuously changes at each step of corrosion, the bare surface is still a robust structural model to study more complex reactions, involving for example the phosphorylation of organic species, like sugars and nitrogenous bases, in order to study the formation of increasingly complex prebiotic molecules which will be the next step to be studied. Overall, these results demonstrate that the pristine schreibersite (001) surface provides an adequate atomistic model for investigating the prebiotic chemistry of phosphorus, avoiding the need for computationally demanding models that explicitly account for advanced stages of surface corrosion.

## Supplementary Material





## Notes

All optimized structures (in Moldraw format) (ZIP)
Tables S1–S3: reporting minima, activation barriers, rate constants, and half-life times of the reaction path of the three (001) schreibersite surface models; Figure S1: showing a bar chart of the same minima and activation barriers (PDF)
