# Supplementary material for: Can Defective Schreibersite Deliver Prebiotic Phosphorus?
Source: ACS Earth Space Chem. 2026 Sep 7;10(9):2045–52. doi: 10.1021/acsearthspacechem.6c00158 (PMC13592078; doi:10.1021/acsearthspacechem.6c00158)
Supplement: Supplementary file 1 [file sp6c00158_si_002.pdf]

Can Defective Schreibersite Deliver Prebiotic Phosphorus?

Supporting Information

Stefano Pantaleone,[1,] Albert Rimola,[2,3] Nadia Balucani,[4,5,6] and Piero Ugliengo[1]*

[1]Dipartimento di Chimica and Nanostructured Interfaces and Surfaces (NIS) Centre, Università degli Studi di Torino, via P. Giuria 7, I-10125, Torino, Italy

[2]Departament de Química, Universitat Autònoma de Barcelona, 08193 Bellaterra, Catalonia, Spain

[3]Accademia delle Scienze di Torino, 10123 Torino, Italy

[4]Dipartimento di Chimica, Biologia e Biotecnologie, Università degli Studi di Perugia, Via Elce di Sotto 8, I-06123 Perugia, Italy

[5]Osservatorio Astrofisico di Arcetri, Largo E. Fermi 5, I-50125 Firenze, Italy

[6]Université Grenoble Alpes, CNRS, Institut de Planétologie et d'Astrophysique de Grenoble (IPAG), F-38000 Grenoble, France

KEYWORDS. DFT, periodic surface, defects, corrosion, prebiotic chemistry

Table S1. Gibbs free reaction energies (at 298 K) and activation barriers (in kJ/mol) and corresponding rate constant (in s^{-1}) and half-life times (in hours) of the water corrosion process on pristine (001) schreibersite surface.

	ΔG	activation barriers	k	$t_{1/2}$
Reag-H_2O	0.0			
TS-OH-H	35.2	35.2	4.18E+06	4.61E-11
INT-OH-H	-51.0			
TS-POH	35.3	86.3	4.62E-03	4.17E-02
INT-POH	2.9			
Reag-$2H_2O$	2.9			
TS-POH-OH	37.8	34.8	4.84E+06	3.98E-11
INT-POH-OH	-93.3			
TS-$P(OH)_2$	-1.9	91.4	5.90E-04	3.26E-01
INT-$P(OH)_2$	-14.5			
Reag-$3H_2O$	-14.5			
TS-$P(OH)_2$-OH	-4.8	9.7	1.26E+11	1.53E-15
INT-$P(OH)_2$-OH	-51.8			
TS-$P(OH)_3$	43.0	94.8	1.48E-04	1.30E+00
Prod-$P(OH)_3$	8.7			
TS-P(O-H)$(OH)_2$	13.5	4.9	8.68E+11	2.22E-16
P(=O)$(OH)_2$	-85.2			
Reag-$4H_2O$	-85.2			
TS-P(=O)$(OH)_2$OH-H	-32.0	53.2	2.95E+03	6.52E-08
P(=O)$(OH)_2$OH-H	-64.8			
TS-H_3PO_4	50.0	114.8	4.78E-08	4.03E+03
Prod-H_3PO_4	-69.2			

Table S2. Gibbs free reaction energies (at 298 K) and activation barriers (in kJ/mol) and corresponding rate constant (in s^{-1}) and half-life times (in hours) of the water corrosion process on defective (001) schreibersite surface.

	ΔG	activation barriers	k	$t_{1/2}$
1H_2O-H_2O	0.0			
1H_2O-DEPT-TS	52.5	52.5	3.86E+03	4.99E-08
1H2O-DEPT	-78.0			
1H_2O-OH-ROT-TS	-66.9	11.1	7.01E+10	2.75E-15
1H_2O-OH-ROT	-80.6			
1H_2O-OH-DIFF-TS	26.2	106.8	1.17E-06	1.65E+02
1H_2O-POH	20.1			
1H_2O-POH-DEPT-TS	26.0	5.9	5.74E+11	3.36E-16
1H_2O-PO-H-H	-85.2			
2H_2O-H_2O	-85.2			
2H_2O-DEPT-TS	-9.0	76.2	2.74E-01	7.02E-04
2H_2O-DEPT	-165.1			
2H_2O-OH-DIFF-TS	-71.9	93.2	2.90E-04	6.63E-01
2H_2O-PO-OH	-79.5			
3H_2O-H_2O	-79.5			
3H_2O-DEPT-TS	-48.6	30.9	2.39E+07	8.04E-12
3H2O-DEPT	-58.0			
3H_2O-DIFF-TS	16.1	74.1	6.49E-01	2.97E-04
3H_2O-POH_3	-46.0			
4H_2O-H_2O	-46.0			
4H_2O-DEPT-TS	-17.5	28.4	6.40E+07	3.01E-12
4H2O-DEPT	-110.4			
4H_2O-POH_3-DEPT-TS	-83.8	26.6	1.34E+08	1.44E-12
4H_2O-POH_3-DEPT	-149.5			
4H_2O-H_3PO_4-TS	-63.8	85.7	6.00E-03	3.21E-02
4H_2O-H_3PO_4	-134.8			

Table S3. Gibbs free reaction energies (at 298 K) and activation barriers (in kJ/mol) and corresponding rate constant (in s^{-1}) and half-life times (in hours) of the water corrosion process on corroded (001) schreibersite surface.

	ΔG	activation barriers	k	$t_{1/2}$
1OH-3OH-H	0.0			
1OH-TS-POH	50.8	50.8	7.78E+03	2.47E-08
1OH-POH	10.4			
2OH-POH-DEPT-TS	91.9	81.5	3.17E-02	6.08E-03
2OH-POH-DEPT	-50.9			
2OH-TS1	-15.7	35.2	4.12E+06	4.67E-11
2OH-INT	-35.2			
2OH-TS2	-5.4	29.8	3.69E+07	5.22E-12
2OH-POOH	-59.7			
3OH-TS1	-21.7	38.0	1.34E+06	1.44E-10
3OH-INT	-42.6			
3OH-TS2	51.2	93.8	2.23E-04	8.64E-01
3OH-PO-OH_2	-47.4			
4OH-H_2O	-47.4			
4OH-DEPT-TS	26.8	74.1	6.34E-01	3.04E-04
4OH-DEPT	-62.9			
4OH-OHROT-TS	-43.3	19.7	2.22E+09	8.69E-14
4OH-OHROT	-44.4			
4OH-ROT-TS	-17.8	26.6	1.37E+08	1.41E-12
4OH-ROT	-61.4			
4OH-H_3PO_4-TS1	50.0	111.4	1.86E-07	1.04E+03
4OH-H_3PO_4-INT	44.2			
4OH-H_3PO_4-TS2	89.3	45.2	7.53E+04	2.56E-09
4OH-H_3PO_4	-34.1			

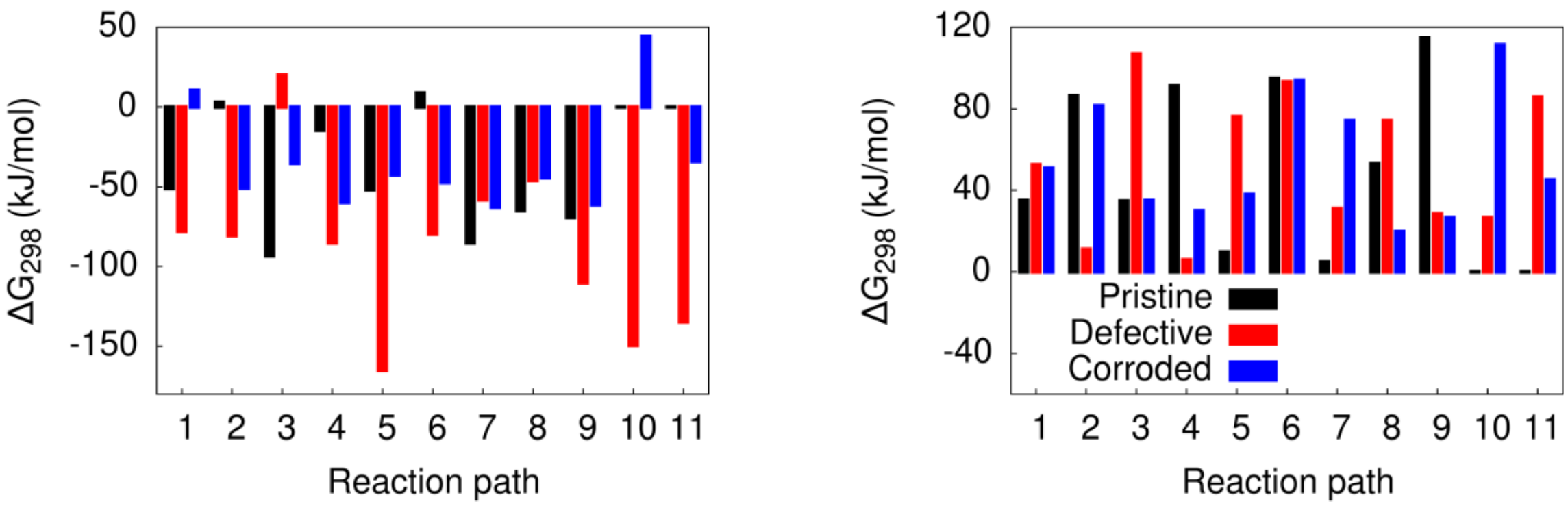

a) Minima **b) Activation barriers**

Figure S1. a) Minima calculated using as reference the first minimum of the reaction path. b) Activation barriers, calculated with respect to the preceding minimum. Black color refers to data on pristine (001) surface, as in Ref.[25], while red and blue on defective and corroded models, as studied in this work.
